# Memory Immune Responses against Pandemic (H1N1) 2009 Influenza Virus Induced by a Whole Particle Vaccine in Cynomolgus Monkeys Carrying Mafa-A1*052∶02

**DOI:** 10.1371/journal.pone.0037220

**Published:** 2012-05-18

**Authors:** Masahiko Arikata, Yasushi Itoh, Masatoshi Okamatsu, Toshinaga Maeda, Takashi Shiina, Keiko Tanaka, Shingo Suzuki, Misako Nakayama, Yoshihiro Sakoda, Hirohito Ishigaki, Ayato Takada, Hideaki Ishida, Kosuke Soda, Van Loi Pham, Hideaki Tsuchiya, Shinichiro Nakamura, Ryuzo Torii, Takeshi Shimizu, Hidetoshi Inoko, Iwao Ohkubo, Hiroshi Kida, Kazumasa Ogasawara

**Affiliations:** 1 Division of Pathology and Disease Regulation, Department of Pathology, Shiga University of Medical Science, Otsu, Japan; 2 Department of Otorhinolaryngology-Head and Neck Surgery, Shiga University of Medical Science, Otsu, Japan; 3 Division of Molecular Medical Biochemistry, Shiga University of Medical Science, Otsu, Japan; 4 Research Center for Animal Life Science, Shiga University of Medical Science, Otsu, Japan; 5 Laboratory of Microbiology, Department of Disease Control, Graduate School of Veterinary Medicine, Hokkaido University, Sapporo, Japan; 6 Research Center for Zoonosis Control, Hokkaido University, Sapporo, Japan; 7 Department of Molecular Life Science, Division of Basic Medical Science and Molecular Medicine, Tokai University School of Medicine, Isehara, Japan; Pasteur Institute of Shanghai, Chinese Academy of Science, China

## Abstract

We made an H1N1 vaccine candidate from a virus library consisting of 144 ( = 16 HA×9 NA) non-pathogenic influenza A viruses and examined its protective effects against a pandemic (2009) H1N1 strain using immunologically naïve cynomolgus macaques to exclude preexisting immunity and to employ a preclinical study since preexisting immunity in humans previously vaccinated or infected with influenza virus might make comparison of vaccine efficacy difficult. Furthermore, macaques carrying a major histocompatibility complex class I molecule, Mafa-A1*052∶02, were used to analyze peptide-specific CD8^+^ T cell responses. Sera of macaques immunized with an inactivated whole particle formulation without addition of an adjuvant showed higher neutralization titers against the vaccine strain A/Hokkaido/2/1981 (H1N1) than did sera of macaques immunized with a split formulation. Neutralization activities against the pandemic strain A/Narita/1/2009 (H1N1) in sera of macaques immunized twice with the split vaccine reached levels similar to those in sera of macaques immunized once with the whole particle vaccine. After inoculation with the pandemic virus, the virus was detected in nasal samples of unvaccinated macaques for 6 days after infection and for 2.67 days and 5.33 days on average in macaques vaccinated with the whole particle vaccine and the split vaccine, respectively. After the challenge infection, recall neutralizing antibody responses against the pandemic virus and CD8^+^ T cell responses specific for nucleoprotein peptide NP262-270 bound to Mafa-A1*052∶02 in macaques vaccinated with the whole particle vaccine were observed more promptly or more vigorously than those in macaques vaccinated with the split vaccine. These findings demonstrated that the vaccine derived from our virus library was effective for pandemic virus infection in macaques and that the whole particle vaccine conferred more effective memory and broader cross-reactive immune responses to macaques against pandemic influenza virus infection than did the split vaccine.

## Introduction

A pandemic (2009) H1N1 influenza A virus has been transmitted among humans since April 2009 [1]. We revealed that the pandemic (2009) H1N1 virus replicated efficiently in non-human primates and caused more severe pathological changes in the lungs of infected macaques than did a circulated human H1N1 (Russian flu) virus [2]. A substantial number of hospitalized individuals did not have underlying health issues during the pandemic [3], [4], and their symptoms were as severe as those seen in cynomolgus macaques [2], [5], [6]. In addition, cynomolgus macaques are susceptible to other unadapted human influenza viruses after minimal passages in cell culture for isolation of the virus [7]. Since the clinical symptoms seen in cynomolgus macaques infected with influenza viruses closely reflect the signs of disease observed in humans, cynomolgus macaque models of influenza virus infection are useful for predicting symptoms and extrapolating pathogenesis in humans. Therefore, we examined the efficacy of vaccines against pandemic (H1N1) 2009 influenza virus using macaques.

In the present study, we selected a vaccine strain from a non-pathogenic influenza A virus library that contains 144 different combinations of 16 hemagglutinins (HA) and 9 neuraminidases (NA) subtypes, and we examined the efficacy of the vaccine [8]–[11], and then compared differences in formulations of vaccines, whole particle vaccines and split vaccines. Although the efficacy of whole particle vaccines has been described previously in humans [12], it is difficult to exclude disturbance of pre-existing immunity due to previous infection with influenza viruses [2], [13], [14]. We used immunologically naïve non-human primates to test the vaccine efficacy with focus on induction of memory cytotoxic T lymphocyte (CTL) responses. In addition, animal models enable examination of the time lag between infection with a virus and initiation of immune responses, which is shorter in recall memory responses than in primary responses. Thus, non-human primates would be excellent tools to examine memory responses after vaccination.

A problem in studies using non-human primates is the difficulty in searching for epitopic peptides in individual animals to analyze peptide-specific T cell responses since major histocompatibility complex (MHC) genes are polymorphic and most of the macaques used for biomedical research are not inbred strains [15]–[18]. To solve this problem and to precisely analyze CTL responses specific for influenza virus peptides in macaques, we used macaques expressing Mafa-A1*052∶02, which was observed at a frequency of 17% in the Mafa-A1 allele of cynomolgus macaques originating from the Philippines (Shiina et al., unpublished data).

To examine peptide-specific memory CTL responses, a Mafa-A1*052∶02- binding motif and epitopes of nucleoprotein (NP) of the pandemic virus were determined using two approaches. Firstly, we used a peptide-binding assay with overlap peptides. These peptides were mixed with cells lacking transporter associated with antigen processing (TAP) proteins, which do not present endogenous cytosolic peptides on MHC class I molecules or do not allow stable expression of MHC class I molecules on the cell surface unless appropriate exogenous peptides are added [19], [20]. Therefore, binding of peptides to MHC class I is detected as stable expression of MHC class I molecules. Secondly, we identified naturally processed peptides eluted from MHC molecules using liquid chromatography with tandem mass spectrometry (LC-MS/MS) and a genetic information database as previously described [21]. Consequently, we determined NP peptides that bound to Mafa-A1*052∶02.

Recall memory CTL responses specific for NP peptides in lymph node cells from macaques immunized with the whole particle vaccine were more vigorous than those in lymph node cells from macaques immunized with the split vaccine. Furthermore, recall neutralization antibody responses against the pandemic challenge strain in macaques vaccinated with the whole particle vaccine were observed more promptly than those in macaques vaccinated with the split vaccine. Therefore, the results suggest that the inactivated whole particle vaccine is more effective against pandemic influenza virus infection than is the split vaccine, inducing cross-reactive memory CTL and antibody responses.

## Results

### Antibody Responses After Inoculation of an Inactivated Whole Virus Particle Vaccine or a Split Vaccine Prepared from a Non-pathogenic H1N1 Strain in a Virus Library

To compare immune responses in different vaccine formulations, we prepared an inactivated whole viral particle vaccine and a split vaccine using the A/swine/Hokkaido/2/1981 (H1N1) (Hokkaido2) strain that was selected from our virus library previously described [8]. The vaccine was subcutaneously inoculated twice into each macaque, and antibody responses in the vaccinated macaques were examined during a 7-week period between the second vaccination and challenge with an H1N1 pandemic virus. The macaques showed no systemic symptoms after the vaccination. No skin reaction at the site of injection was observed after inoculation with either the whole particle vaccine or split vaccine.

**Figure 1:**
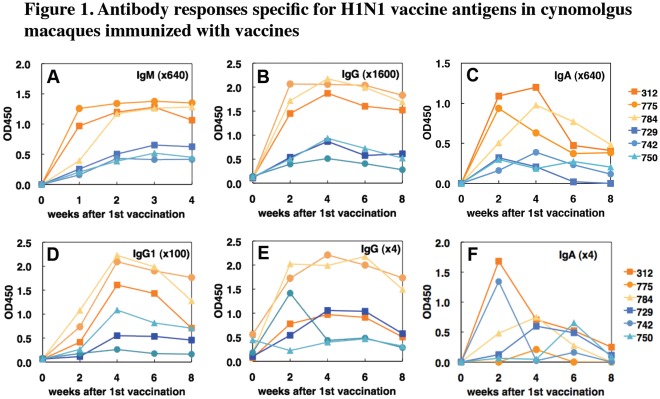
Antibody responses specific for H1N1 vaccine antigens in vaccinated cynomolgus macaques. Cynomolgus macaques were subcutaneously immunized twice (in weeks 0 and 2) with a whole virus particle vaccine (#312, #775 and #784, orange symbols) or with a split vaccine (#729, #742 and #750, blue symbols) derived from Hokkaido2. Sera and swab samples were collected in indicated weeks after the first vaccination. IgM (A), IgG (B, E), IgA (C, F), and IgG1 (D) antibodies specific for Hokkaido2 antigens in sera (A-D) and nasal swab samples (E, F) were analyzed using ELISA. Optical densities at 450 nm are shown. In IgM and IgA measurements (A, C, F), the OD450 values before vaccination as non-specific background responses were subtracted from those after vaccination.

Serum IgM specific for Hokkaido2 antigen was clearly detected in 2 of the 3 macaques vaccinated with the whole particle vaccine one week after the first vaccination and in all macaques vaccinated with the whole particle vaccine 2 weeks after the first vaccination (before the second vaccination that was performed 2 weeks after the first vaccination) (Fig. 1A). In the 3 macaques vaccinated with the whole particle vaccine, serum total IgG, IgA, and IgG1 specific for Hokkaido2 were detected 2 weeks after the first vaccination and production of IgG1 antibody was enhanced after the second vaccination (Fig. 1B–D). Antigen-specific antibody levels gradually declined from 2 weeks after the second vaccination. On the other hand, serum IgM, IgG, IgA and IgG1 responses specific for Hokkaido2 in macaques vaccinated with the split vaccine were lower than those in macaques vaccinated with the whole particle vaccine (Fig. 1A–D). Antigen-specific IgG antibodies in nasal swab samples from all of the macaques vaccinated with the whole particle vaccine and 2 of the 3 macaques vaccinated with the split vaccine (except #750) were detected 2 weeks after the first vaccination (Fig. 1E). Antigen-specific IgA was detected in swab samples from 2 macaques vaccinated with the whole particle vaccine (#312 and #784) and in swab samples from all 3 macaques vaccinated with the split vaccine (Fig. 1F).

**Figure 2:**
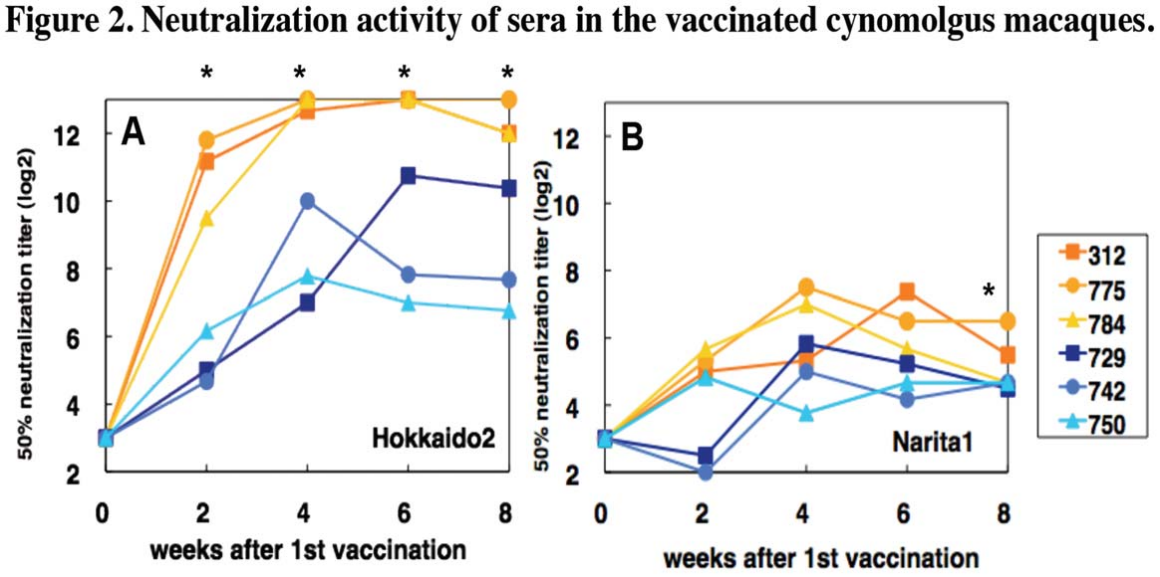
Neutralization activity of sera in the vaccinated cynomolgus macaques. Sera were collected in the indicated weeks after the first vaccination. Sera of week 2 were obtained before the second vaccination. The neutralization titers against Hokkaido2 (A) and Narita1 (B) were expressed as reciprocals of dilution of the serum samples that showed CPE in 50% of the wells. Detection limits were 1∶8 in week 0 and 1∶4 in the other weeks. P values calculated with Student’s *t*-test are less than 0.05 in comparison between the whole particle vaccine group and split vaccine group against Hokkaido2 and Narita1 (*) when the titers below 1∶4 are calculated as 1∶4.

Next, we examined neutralization activity of serum antibody from macaques vaccinated with the whole particle or the split vaccine. Sera from macaques vaccinated with the whole particle vaccine showed significantly higher neutralization activity against Hokkaido2 than did sera from macaques inoculated with the split vaccine from week 2 to week 8 (P<0.05) (Fig. 2A) and less potent neutralization activity against the pandemic strain A/Narita/1/2009 (H1N1) pdm (Narita1) [22], [23] than against Hokkaido2 (Fig. 2B). Sera from two macaques vaccinated with the split vaccine 2 weeks after the first vaccination showed no detectable neutralization activity against Narita1. Sera from two macaques (#729, #742) vaccinated with the split vaccine 2 weeks after the second vaccination (week 4) showed neutralization activity against Narita1 at a level comparable to that in sera from macaques vaccinated once with the whole particle vaccine (week 2). The average of 50% neutralization titers against Narita1 in sera from macaques vaccinated with the whole particle vaccine was significantly higher than that in sera from macaques vaccinated with the split vaccine in week 8 (P<0.05) (Fig. 2B). These results suggested that a single vaccination with a whole particle vaccine has potency similar to that of two inoculations with split vaccines in serum neutralization activity.

### T lymphocyte Responses Specific for Vaccine Antigens After the Second Vaccination in Cynomolgus Macaques

**Figure 3:**
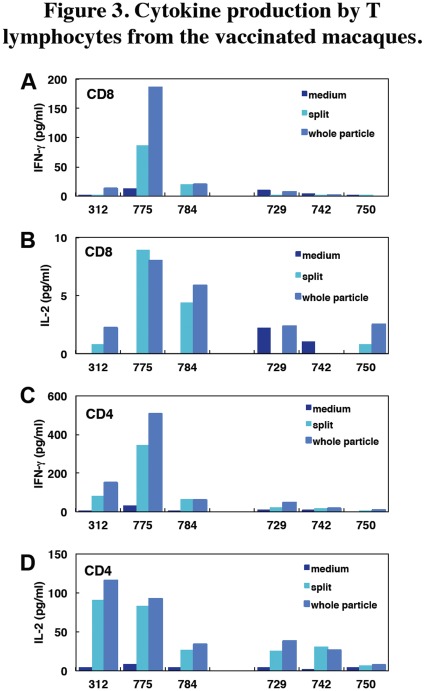
Cytokine production by T lymphocytes from the vaccinated macaques. CD8^+^ cells (A, B) and CD4^+^ cells (C, D) were separated from the blood cells 2 weeks after the second vaccination (week 4). T lymphocytes were cultured with irradiated APC and the whole particle or split vaccine antigen (10 µg/ml) for 48 h. Culture without antigen is indicated as medium. Cytokines (IFN-γ; A, C, IL-2; B, D) in the supernatants were measured with a multiple cytokine array. IL-4 production was under the detection limit.

Two weeks after the second vaccination (week 4), cytokine production by T lymphocytes specific for the vaccine antigen was analyzed. CD4^+^ and CD8^+^ T cells from all macaques vaccinated with the whole particle vaccine produced interferon (IFN)-γ and interleukin (IL)-2 (Fig. 3). In contrast, T cells from macaques vaccinated with the split vaccine produced smaller amounts of IFN-γ and IL-2 than did those from macaques vaccinated with the whole particle vaccine even when cells were stimulated with the whole particle antigen, though the differences were not statistically significant. These results indicated that subcutaneous vaccinations with the whole particle vaccine induced greater T cell responses, especially Th1 type responses in both CD4^+^ and CD8^+^ T cells, than did the split vaccine, as well as IgG1 antibody responses (Fig. 1), which are assisted by Th1 cells in humans.

### Protective Effects of Two Formulations of the Inactivated Vaccine Against a Pandemic (H1N1) 2009 Strain in Cynomolgus Macaques

**Figure 4:**
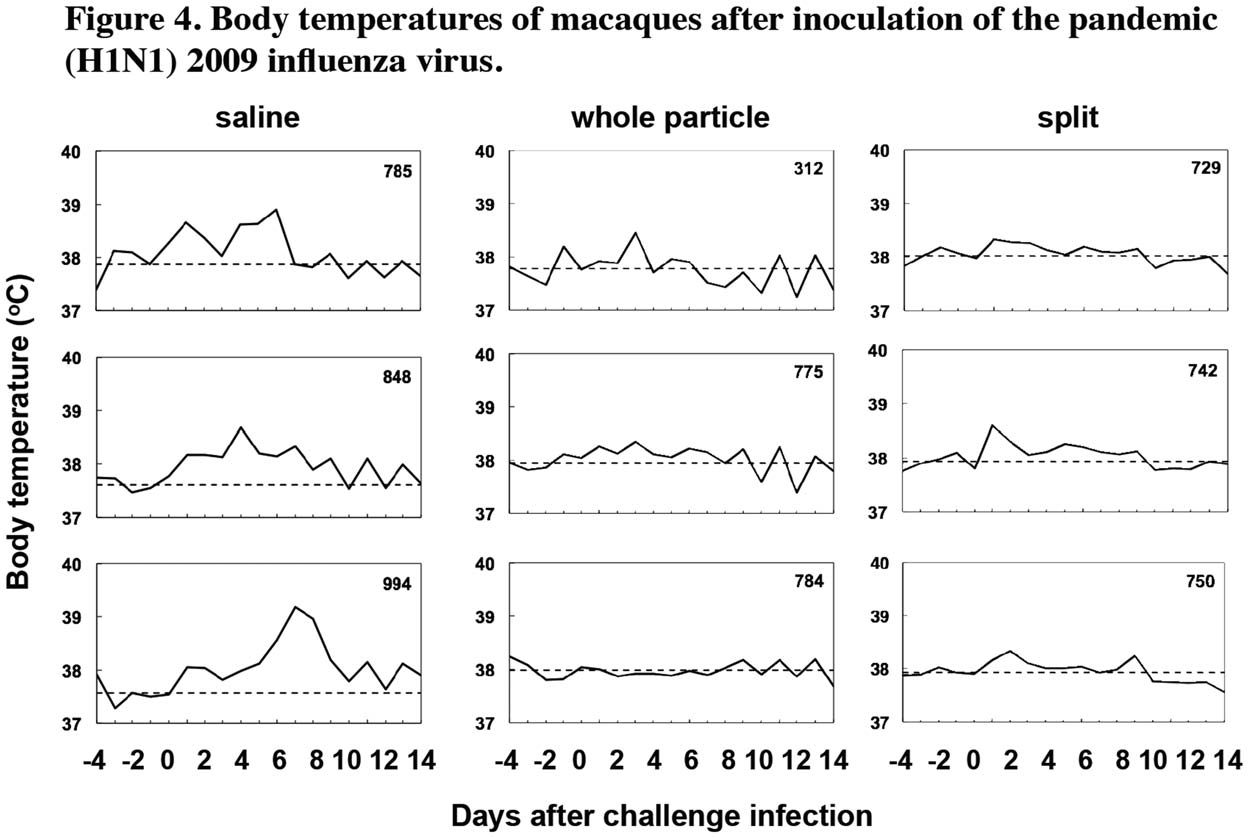
Body temperatures of macaques after inoculation of the pandemic (H1N1) 2009 influenza virus. Cynomolgus macaques were subcutaneously immunized with the whole particle vaccine (middle column) or with the split vaccine (right column). Cynomolgus macaques injected with saline were used as unvaccinated controls (left column). On day 0, 7 weeks after the second vaccination, Narita1 (2×10^5^ TCID_50_) was inoculated into nasal cavities of the macaques. Lines horizontally drawn indicate average temperature levels at pre-infection.

Next, to examine efficacy of the vaccines, the pandemic (H1N1) 2009 virus strain, Narita1, was inoculated into nasal cavities of macaques 7 weeks after the second vaccination. Body temperature was expressed by calculating the average of the highest and lowest temperatures in one day, and body temperature after the virus challenge was compared with that before the virus challenge. When we focused on temperature changes until day 7 after infection, higher body temperature than that before the challenge was observed for 7 to 8 days after infection in the unvaccinated macaques (7.67 days on average, Fig. 4, left panels), while raised body temperatures in the macaques vaccinated with the whole particle vaccine and in macaques vaccinated with the split vaccine were observed for 2 to 8 days (5 days on average, Fig. 4, middle panels) and 6 to 7 days (6.33 days on average, Fig. 4, right panels), respectively, though differences among the three groups were not statistically significant. Therefore, these results indicated a tendency for vaccination with inactivated Hokkaido2 to accelerate recovery of body temperature and reduce morbidity caused by infection with the pandemic influenza virus. No macaques either with or without vaccination lost weight or appetite after inoculation with Narita1 (data not shown).

**Figure 5:**
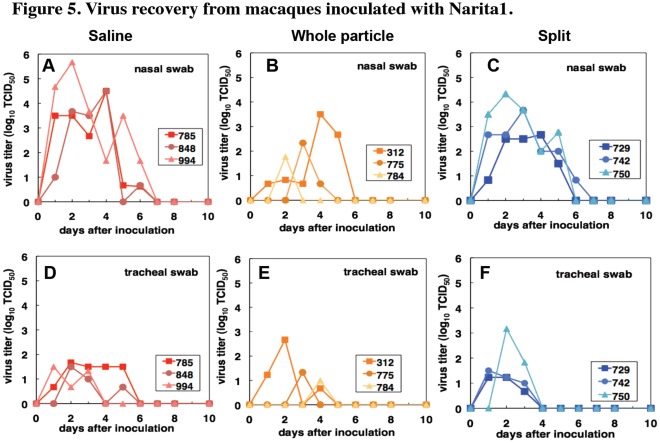
Virus recovery from macaques inoculated with Narita1. Each macaque was inoculated with Narita1 on day 0 (saline: A, D; whole particle vaccine, B, E; split vaccine, C, F). Virus titers in nasal (A-C) and tracheal (D-F) swab samples were determined using MDCK cells. Virus titers were under detection limit after day 7.

We examined the virus titers in swab samples after challenge with the pandemic virus Narita1. The virus was detected in swab samples from nasal cavities of the unvaccinated macaques for 6 days (6 days on average) and in swab samples from tracheas for 3 to 5 days (4.33 days on average) after inoculation with Narita1 (Fig. 5A, D). On the other hand, in the macaques vaccinated with the whole particle vaccine, the virus was detected until day 5 (3.67 days on average) in nasal samples and until day 4 (3.67 days on average) in tracheal samples (Fig. 5B, E). The virus in nasal and tracheal samples of macaques vaccinated with the split vaccine was detected until day 6 (5.33 days on average) and until day 3 (3 days on average), respectively (Fig. 5C, F). The average areas under the curves (AUC) of virus titers were 16.6, 4.37 and 13.4 log_10_TCID_50_/ml•day in nasal samples and 4.50, 2.30 and 3.95 log_10_TCID_50_/ml•day in tracheal samples of macaques inoculated with saline, the whole particle vaccine and the split vaccine, respectively. Significant differences of virus titer AUC in nasal samples were observed between the saline group and whole particle vaccine group (P = 0.016) and between the whole particle vaccine group and split vaccine group (P = 0.03). Therefore, inoculation with the whole particle vaccine of Hokkaido2 derived from the non-pathogenic virus library interfered with propagation of the pandemic strain Narita1 in the upper respiratory tracts more effectively than did inoculation with the split vaccine.

### Memory Antibody Responses after Challenge Infection with the Pandemic Influenza Virus

**Figure 6:**
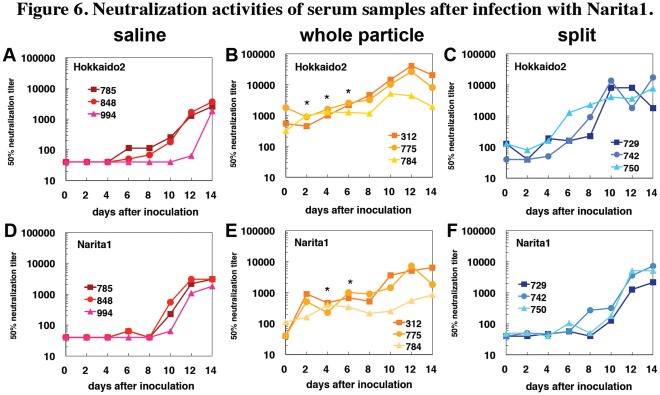
Neutralization activities of serum samples after infection with Narita1. Cynomolgus macaques were vaccinated with saline (A, D), whole particle vaccine (B, E), or split vaccine (C, F) as described in the legend to Fig. 1. Macaques were inoculated with Narita1 as described in the legend to Fig. 4. Sera were collected on indicated days after the challenge infection. Fifty percent neutralization titers against Hokkaido2 (A-C) and Narita1 (D-F) were determined. Statistically significant differences of average neutralization titers between the whole particle vaccine group and split vaccine group are indicated with asterisks (P<0.05 with Student’s *t*-test).

We examined recall antibody responses against the vaccine strain Hokkaido2 and the pandemic strain Narita1 after challenge infection. In the sera from macaques without vaccination, neutralization activity against Hokkaido2 and Narita1 was detected 6 to 10 days after challenge infection with Narita1 (Fig. 6A, D). Neutralization activity against Hokkaido2 and Narita1 was observed in the sera from macaques vaccinated with the whole particle vaccine before the challenge infection (day 0) (Fig. 6B, E), and neutralization titers against Narita1 were increased 2 days after the challenge, suggesting that the challenge infection induced immediate recall antibody responses against Narita1.

Low neutralization activity in sera from macaques vaccinated with the split vaccine was observed against Hokkaido2 but not against Narita1 before the challenge infection, and neutralization activity against Hokkaido2 was increased on days 4 to 6 after the challenge (Fig. 6C, F). On the other hand, neutralization activity against Narita1 was increased on days 8 to 10 in sera from macaques vaccinated with the split vaccine and was significantly lower on day 4 and day 6 than that with the whole particle vaccine (P<0.05). These responses were similar to responses observed in the unvaccinated macaques. These findings indicate that vaccination with the whole particle vaccine generated immunological memory in B cells that produced antibodies against not only the vaccine strain but also the challenge strain (i.e., crossreaction), whereas vaccination with the split vaccine induced memory B cell responses against the vaccine antigens but not against the antigens of the challenge strain.

### Identification of Nucleoprotein Peptides that Induced Mafa-A1*052∶02-restricted CD8^+^ T cell Responses After Challenge with the Pandemic (H1N1) 2009 Strain

**Figure 7:**
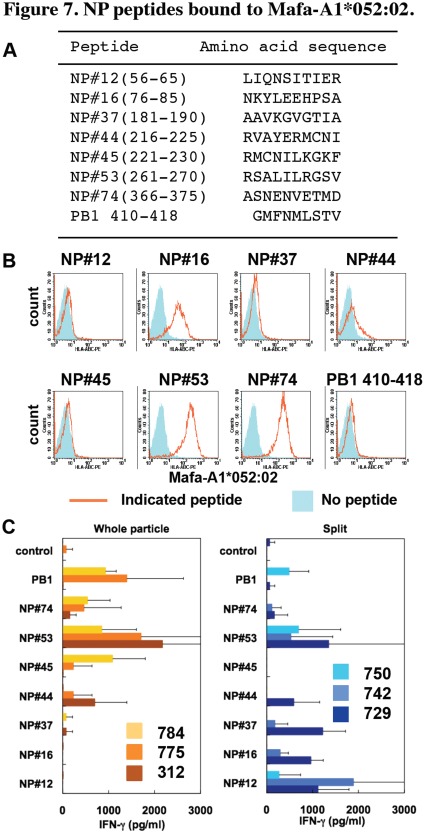
NP peptides bound to Mafa-A1*052∶02. (A) Amino acid sequences of 10-mer peptides derived from Narita1 nucleoprotein are shown as one-letter code. The sequence of influenza A virus PB1 peptide was used as a representative of non-binding peptides to Mafa-A1*052∶02. The numbers in parentheses indicate residues from the N-terminus of NP. (B) RMA-S expressing Mafa-A1*052∶02 and human β2-microglobulin was cultured with NP peptides at 27°C for 1 h and then at 37°C for 4 h. Stable expression of Mafa-A1*052∶02 on the cell surface was examined by staining with W6/32 antibody. Orange lines: cells cultured with indicated peptides. Blue shade: cells cultured without peptide. (C) CD8^+^ cells were isolated from spleens of indicated macaques and cultured with APC and indicated peptides. IFN-γ production was determined using ELISA. Averages and SD of triplicate culture are shown. Representative results of three independent experiments are shown.

Next, we examined memory CTL responses in macaques vaccinated with the whole particle vaccine and the split vaccine. To measure antigen-specific CTL responses, we used macaques carrying an MHC class I molecule, Mafa-A1*052∶02 (Table 1). Furthermore, we prepared 10-mer influenza virus peptides derived from NP, which overlapped 5 amino acids in consecutive peptides and showed amino acid sequences of some peptides since NP was shown in mice to be a nuclear/cytosolic target antigen for CTL (Fig. 7A). Firstly, we determined peptides bound to Mafa-A1*052∶02. To perform a peptide binding assay, we established an RMA-S cell line that lacked TAP-2 protein for presentation of endogenous peptides and expressed Mafa-A1*052∶02 and human β2-microglobulin for presentation of exogenous peptides. Using the RMA-S transfectant, we found that 3 NP peptides (#16, #53 and #74) bound to Mafa-A1*052∶02 (Fig. 7B). NP#44 showed a weak binding capacity to Mafa-A1*052∶02 on the surface of the RMA-S transfectant.

**Table 1:** Mafa-A1 alleles of macaques used in the present study.

	Mafa-A1	
Animal ID	Allele 1	Allele 2
785	052∶02	089∶03
848	052∶02	089∶03
994	052∶02	008∶02
312	052∶02	004∶01
775	052∶02	093∶01
784	052∶02	089∶03
729	052∶02	094∶01
742	052∶02	093∶02
750	052∶02	094∶01

We cultured CD8^+^ T cells from Narita1-infected macaques with the NP peptides and measured IFN-γ responses. IFN-γ production was detected in the culture of CD8^+^ T cells from macaques vaccinated with the whole particle vaccine with NP#44, #45, #53 and #74 and from macaques vaccinated with the split vaccine with NP#12, #16, #37, #44, #53 and #74 (Fig. 7C).

**Figure 8:**
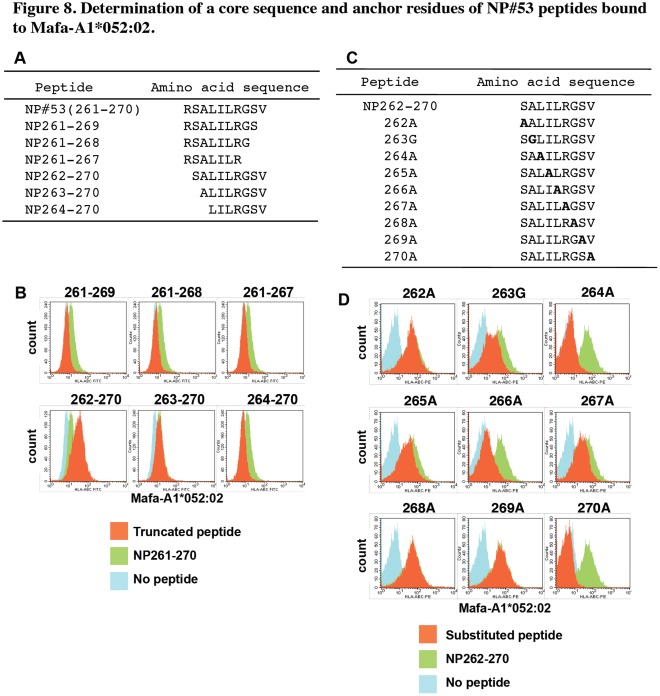
A core sequence and anchor residues of NP#53 peptides bound to Mafa-A1*052∶02. (A) Amino acid sequences of truncated NP#53 peptides. (B) Truncated NP#53 peptides were cultured with the Mafa-A1*052∶02 transfectant as described in the legend to Fig. 7. Orange: cells cultured with truncated NP#53 indicated in each histogram. Green: cells cultured with untruncated NP#53 (261–270). Blue: cells cultured without peptide. (C) Amino acid sequences of substituted NP262-270 peptides. Amino acids of NP262-270 were substituted to alanine except residue 263 that was substituted to glycine. (D) Substituted NP262-270 peptides were cultured with the Mafa-A*052∶02 transfectant. Orange: cells cultured with substituted NP262-270 indicated in each histogram. Green: cells cultured with unsubstituted NP262-270. Blue: cells cultured without peptide. Representative results of three independent experiments are shown.

Since IFN-γ production by stimulation with NP#53 was observed in the culture of cells from all vaccinated macaques (Fig. 7C), we performed further analyses of the NP#53 peptide. A peptide lacking C-terminal residue 270 (NP261-269) lost binding capacity to Mafa-A1*052∶02 (Fig. 8A, B), suggesting that the residue 270 V was one of the anchor residues. Furthermore, deletion of residue 261 enhanced binding of the peptide (NP262-270) to Mafa-A1*052∶02, and NP263-270 showed binding to Mafa-A1*052∶02 similar to that of NP261-270, suggesting that NP262-270 was an optimal sequence bound to Mafa-A1*052∶02. Henceforth, we used NP262-270 as a basic peptide bound to Mafa-A1*052∶02. Deletion of residue 263 impaired binding to the Mafa-A1*052∶02 transfectant, indicating that position 263 was one of the anchor residues or that it affected binding capacity.

To determine the precise anchor residues, we used a series of peptides of which amino acids were substituted (Fig. 8C). Substitutions at residues 264, 266 and 270 diminished binding of peptides to Mafa-A1*052∶02 (Fig. 8D), suggesting that residues 264 (position 3 from the N-terminus), 266 (position 5) and 270 (position 9) were anchor residues that were essential for presentation on Mafa-A1*052∶02 and that L at position 3, L at position 5 and V at position 9 comprised a binding motif for Mafa-A1*052∶02. Substitutions at residues 263, 265 and 267 had weak effects on the binding of peptides to Mafa-A1*052∶02.

We examined sequences of naturally processed peptides bound to Mafa-A1*052∶02 using a 721.221 transfectant established by introduction of a Mafa-A1*052∶02 gene into an MHC null human B cell line and LC-MS/MS [21]. In analyses of peptides eluted from Mafa-A1*052∶02 with 9 to 12 amino acids in length, hydrophobic residues were observed at position 9 when C-terminal residues were aligned as position 9 (Table 2). In addition, leucine or isoleucine was detected at position 5 in 8 of the 15 peptides, and leucine, isoleucine or valine was detected at position 3 in 4 of the 15 peptides. This motif was compatible with the sequence of NP262-270 (SALILRGSV).

**Table 2:** Amino acid sequences of peptides eluted from Mafa-A1*052∶02.

sources	NCBI accession	start position	MW^1^	amino acid sequences^2^											
	GI number						1	2	3	4	5	6	7	8	9^3^
Guanylate cyclase soluble subunit α3 isoform A	67763816	127	1112.66			V	P	V	E	V	**I** 4	K	E	S	**L**
S-adenosylmethionine synthase isoform type 2	5174529	150	1235.75		M	P	L	T	**I**	V	**L**	A	H	K	**L**
proliferation-associated protein 2G4	124494254	180	1225.62		T	P	I	E	G	M	**L**	S	H	Q	**L**
tudor domain containing 7	112293287	975	1296.84	R	V	L	L	K	G	I	**L**	T	N	G	**L**
FLJ00043 protein/EH domain-binding protein 1-like protein-1	150378549	904	980.59				A	P	**V**	T	Q	P	R	V	**L**
CAP-Gly domain-containing linker protein1/2	4506751	273/280	1250.77		A	P	I	H	K	V	**I**	R	I	G	**F**
SEC14-like protein 1	221316682	206	1238.74	V	V	I	P	E	A	A	**L**	K	E	G	**L**
60 S ribosomal protein L15	15431293	83	1403.79	K	P	V	H	H	G	V	N	Q	L	K	**F**
structural maintenance of chromosomes flexible hinge domain-containing protein 1	148839305	807	1156.72			R	P	L	P	S	K	A	I	K	**F**
mitotic checkpoint protein BUB3	4757880	11	1303.65	Q	P	P	E	D	G	I	S	S	V	K	**F**
dnaJ homolog subfamily A member 1	4504511	313	1520.86	R	P	Y	E	K	G	R	**L**	I	I	E	**F**
FH1/FH2 domain-containing protein 1	118572599	107	1182.76			K	P	T	**L**	I	**L**	R	T	Q	**L**
intraflagellar transport protein 57 homolog	8922256	100	1193.56				R	P	F	E	Q	P	Q	E	**Y**
ubiquitin-conjugating enzyme E2 D4	8393719	43	1001.47				S	P	Y	Q	G	G	V	F	**F**
vacuolar protein sorting-associated protein 13A	66346674	2184	1043.42				T	C	**V**	T	S	I	C	E	**M**

1 Molecular weight of eluted peptides.

2 Amino acid sequences of peptides eluted from Mafa-A1*052∶02 are shown as a one-letter code.

3 C-terminals of peptides are aligned as position 9.

4 Bold letters are amino acids that are compatible with the predicted Mafa-A1*052∶02-binding motif.

### Memory T Cell Responses After Challenge with the Pandemic (H1N1) Strain in Vaccinated Macaques

**Figure 9:**
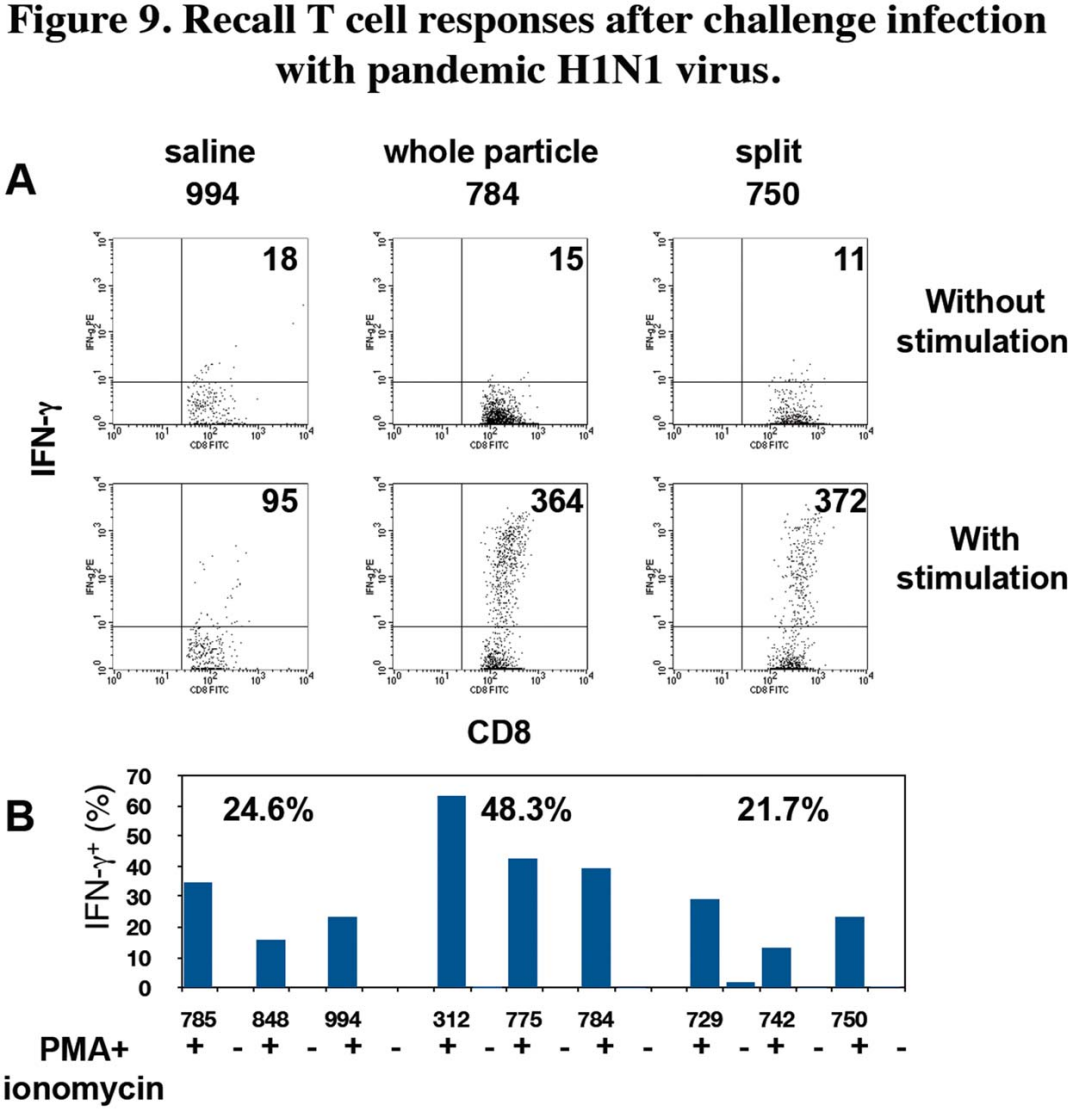
Recall T cell responses after challenge infection with pandemic H1N1 virus. Cervical lymph node cells from the macaques 14 days after challenge with the pandemic H1N1 virus were cultured with NP262-270 peptide for 5 d. The recovered cells were stimulated with (+) or without (-) PMA and ionomycin for 5 h. Thereafter, intracellular IFN-γ and surface CD8 were stained. (A) Representative IFN-γ and CD8 profiles of each group are shown after gating of CD8^+^ cells. Upper figures: culture without PMA and ionomycin, lower figures: culture with PMA and ionomycin. Mean fluorescence intensity (MFI) of IFN-γ staining is indicated in each figure. The averages of MFI of IFN-γ^+^ CD8^+^ cells stimulated with PMA and ionomycin are 147, 395 and 285 in the saline group, the whole particle vaccine group and the split vaccine group, respectively. (B) The percentages of IFN-γ^+^ cells in CD8^+^ cells are shown. The averages of percentages of three macaques in each group are indicated in the graph. There are significant differences (P<0.05) between the saline group and whole particle vaccine group and between the whole particle vaccine group and split vaccine group.

Finally, we examined CD8^+^ T cell responses against NP262-270 using cervical lymph node cells of the infected macaques. IFN-γ production in CD8^+^ T cells was observed after culture with NP262-270 and restimulation (Fig. 9A). The percentage of IFN-γ-producing cells in CD8^+^ T cells stimulated *in vitro* with NP262-270 from macaques vaccinated with the whole particle vaccine (48.3% on average) was significantly higher than the percentage of those from macaques vaccinated with saline (24.6% on average, P = 0.034 in saline vs. whole particle vaccine) and with the split vaccine (21.7% on average, P = 0.049 in whole particle vaccine vs. split vaccine) (Fig. 9B). Since IFN-γ production in CD8^+^ T cells from the macaques inoculated with saline was the primary response with Narita1 infection, the higher percentage of IFN-γ-producing CD8^+^ T cells in the whole particle group than that in the saline group seemed to indicate memory responses induced by vaccination. These responses were likely to be memory recall responses since the recall responses in macaques inoculated with the whole particle vaccine were examined 7 weeks after the vaccination. In contrast, there was no significant difference in the percentage of IFN-γ-producing CD8^+^ cells between the saline group and split vaccine group (Fig. 9B), though IFN-γ production level per cell (mean fluorescense intensity) in the split vaccine group was higher than that in the saline group (Fig. 9A). These results indicated that the whole particle vaccine more effectively generated memory T cells than did the split vaccine.

## Discussion

We examined antibody and T cell responses induced by subcutaneous vaccination with two forms of preparation, whole particle vaccine and split vaccine, using immunologically naïve cynomolgus macaques for influenza virus infection. The macaque model enabled evaluation of vaccine efficacy without consideration of pre-existing immunity, which has been difficult to exclude in human studies [2], [13], [14]. Since most inactivated vaccines are inoculated subcutaneously or intramuscularly in humans [14], [24], we subcutaneously inoculated vaccines into macaques to reflect results in the present study to human vaccination usage. As a result, the whole particle vaccine derived from our virus library was more effective for conferring memory immune responses and prohibiting virus replication than was the split vaccine.

We previously revealed that pandemic (H1N1) 2009 influenza virus caused viral pneumonia in cynomolgus macaques, which resembled pneumonia in human patients during the pandemic [2]–[6]. Furthermore, we were able to analyze immune responses in macaques using antibodies against human molecules [25], whereas reagents for immunological analyses were not sufficient in ferrets that showed human-like clinical symptoms by human influenza viruses. Therefore, we considered the macaque models to be suitable for extrapolation of responses and efficacy of vaccines against pandemic influenza virus infection in humans.

As shown in Fig. 7C, CD8^+^ T cells of individual vaccinated macaques responded to different sets of NP peptides since the macaques used in the present study were not inbred. To compare peptide-specific T cell responses between two vaccinations, we required a parameter shared by all studied macaques. Therefore, we selected macaques carrying Mafa-A1*052∶02 and identified NP262-270 as a peptide that induced CD8^+^ T cell responses in all vaccinated macaques. The variety of stimulatory peptides except NP262-270 among macaques might be due to antigen presentation on other Mafa class I molecules, i.e., the second allelic product of Mafa-A1 other than Mafa-A1*052∶02 and Mafa-B (Table 1). Therefore, we examined CD8^+^ T cell responses specific for NP262-270 plus Mafa-A1*052∶02 to compare memory T cell responses in macaques vaccinated with the whole particle vaccine with those in macaques vaccinated with the split vaccine.

One of the peptides that bound to Mafa-A1*052∶02, NP#74, contained a known CTL epitope (ASNENVETM, NP366-374) bound to mouse MHC class I H-2D^b^, in which N at position 5 and M at position 9 are known as main anchor residues and N at position 3 is a minor anchor [26]. The results presented in Fig. 8 showed that anchor positions of NP262-270 were aligned with the same intervals as those seen in NP366-374 bound to H-2D^b^. Therefore, it is possible that the Mafa-A1*052∶02 binding motif is similar to that of H-2D^b^, i.e., hydrophobic or non-polar residues at positions 3, 5 and 9. This was supported by analyses of peptides eluted from Mafa-A1*052∶02 (Table 2). Based on these results, the Mafa-A1*052∶02 binding motif appeared to be hydrophobic residues (L, I or V) at position 3, hydrophobic residues (L or I) at position 5 and hydrophobic or aromatic residues (L, V, M, F or Y) at position 9 as anchors.

Using macaques carrying Mafa-A1*052∶02, we showed advantages of the whole particle vaccine in three aspects, immunological memory, immunogenicity and cross-reactivity. Firstly, we proved the advantage of the whole particle vaccine in memory CTL responses. A higher percentage of IFN-γ-positive CD8^+^ cells specific for the NP262-270 peptide was observed in macaques vaccinated with the whole particle vaccine than in macaques without vaccination and macaques vaccinated with the split vaccine (Fig. 9). Therefore, it is thought that memory T cells were maintained in macaques inoculated with the whole particle vaccine and expanded promptly after challenge infection.

The whole particle vaccine also induced immunological memory in antibody responses more effectively than did the split vaccine. Neutralizing activity of sera against the challenge strain increased 2 days after the challenge infection in macaques vaccinated with the whole particle vaccine, whereas increase of neutralization activity against the challenge strain in sera from macaques vaccinated with the split vaccine was detected on days 8 to 10 after challenge infection (Fig. 6). This was only 2 days earlier than that in macaques without vaccination. Therefore, the whole particle vaccine generated immunological memory in both cellular and humoral responses more effectively than did the split vaccine. Since recall memory B cell responses were impaired in MyD88-deficient mice, Toll-like receptors (TLR) stimulated by whole particle vaccines would play a critical role in generation of memory B cells [27].

Secondly, as previously shown in mouse and human studies [28]–[30], the whole particle vaccine induced higher titers of neutralizing antibody in macaques than did the split vaccine (Fig. 2). This indicates that inactivated whole viral particles were more immunogenic than soluble split antigens in macaques. Several studies have indicated that a virus-like particle (VLP) antigen was superior to a soluble protein in inducing Th1 and CTL responses with effective antigen cross-presentation by dendritic cells [31]–[33]. We also showed that one immunization with the whole particle vaccine induced neutralizing antibody at a level similar to that induced by two immunizations with the split vaccine. This result suggests that we could reduce the dose of vaccination by using whole particle vaccines to save vaccine amounts and distribute vaccines to more people than by using split vaccines.Not only formulation of antigens but also additional molecules included in the whole particle vaccines were thought to affect the immunogenicity. IL-12 and tumor necrosis factor (TNF)-α production by human dendritic cells was enhanced by whole particle vaccines but not by subunit vaccines [34]. This response might be partly mediated by TLR signals [35]. Inactivated whole virus particles showed adjuvant effects by activating the TLR7-MyD88 pathway in plasmacytoid dendritic cells [36]. Type I interferon production by plasmacytoid dendritic cells was crucial for induction of primary B cell and CD4^+^ T cell responses and cross-presentation of antigen by dendritic cells, resulting in polarization to Th1 cells and activation of CTL [37], [38]. The TLR signal also stimulated naïve B cells directly and enhanced B cell responses [39], [40]. Concordant with the previous reports, we showed that IgG1 responses, one of the hallmarks of Th1 responses in human immune responses, were induced more effectively in cynomolgus macaques after vaccination with the whole particle vaccine than with the split vaccine (Fig. 1), indicating that whole particle vaccines including virus RNA as a natural adjuvant enhanced immunogenicity and induced Th1 responses [28], [41], [42]. Therefore, whole particle vaccines do not necessarily need inoculation with an additional adjuvant.

Thirdly, sera from macaques inoculated with the whole particle vaccine showed neutralizing activity against not only the vaccine strain but also the challenge strain (Figs. 2 and 6), resulting in lower virus titers after challenge in macaques vaccinated with the whole particle vaccine than in macaques vaccinated with the split vaccine (Fig. 5). In the present study, similarities of HA and NA between the vaccine strain and the challenge virus strain were 89% and 83% at the amino acid level, respectively. These results indicated that whole particle vaccines induced not only a larger amount of specific antibody but also a more broadly cross-reactive antibody than did split vaccines [43]. This might be explained by results of previous studies showing that whole particle vaccines and split vaccines activated different B cell repertoires (clones) [44] and that two formulations of vaccines induced differentiation of distinct T helper cells such as Th1 and Th2 as discussed above, resulting in differences in the number of reacting B cells and affinity maturation [28], [45]. Furthermore, since sequences of NP including CTL epitopes were conserved in influenza A viruses compared with HA and NA, CTL specific for NP and other internal proteins might react to heterosubtypic influenza viruses [46], [47]. Thus, whole particle vaccines would be effective even if viral antigens were changed by gene mutations within a certain range. Therefore, our virus library containing 144 combinations of 16 HA and 9 NA would be useful when pandemic vaccines are prepared as whole particle vaccines to induce both antibody and CTL responses with broad cross-reactivity [8].

In summary, we showed that inactivated whole virus particles from the virus library were effective against pandemic (H1N1) 2009 influenza virus infection using the cynomolgus macaque model. Whole particle vaccines were superior for induction of memory antibody and CTL responses, which were confirmed at the level of peptide-specific responses. We are examining other Mafa class I and class II types and we are breeding macaques carrying specific MHC haplotypes. These macaques will be useful for evaluation of vaccination and analysis of both cellular and humoral immune responses in future studies.

## Materials and Methods

### Animals and Ethics Statement

?This study was carried out in strict accordance with the Guidelines for the Husbandry and Management of Laboratory Animals of Research Center for Animal Life Science at Shiga University of Medical Science and STANDARDS RELATING TO THE CARE AND MANAGEMENT, ETC. OF EXPERIMENTAL ANIMALS (Notification No.6, March 27, 1980 of the Prime Minister’s Office, Japan). The protocol was approved by the Shiga University of Medical Science Animal Experiment Committee (Permit number: 2009-5-2H) and the Biosafety Committee (Permit number: 2009-2). The animal experiments were conducted in strict compliance with animal husbandry and welfare regulations. All procedures were performed under ketamine and xylazine anesthesia, and all efforts were made to minimize suffering. Food pellets of CMK-2 (CLEA Japan, Inc., Tokyo, Japan) fed once a day after recovery from anesthesia and drinking water were available *ad libitum.* Animals were singly housed under controlled conditions of humidity (40±5%), temperature (25±1°C), and light (12 h light/12 h dark cycle, lights on at 8∶00 A.M.). Five- to seven-year-old female cynomolgus macaques from the Philippines (Ina Research Inc., Ina, Japan) were used. The cynomolgus macaques used in the present study were healthy young adults. In the text and figures, individual macaques are distinguished by identification numbers. The absence of influenza A virus NP-specific antibodies in their sera was confirmed before experiments using an antigen-specific enzyme-linked immunosorbent assay (ELISA), AniGen AIV Ab ELISA (Animal Genetics Inc., Kyonggi-do, Korea), for currently circulating influenza virus. Two weeks before virus inoculation, a telemetry probe (TA10CTA-D70, Data Sciences International, St. Paul, MN) was implanted in the peritoneal cavity of each macaque under ketamine/xylazine anesthesia followed by isoflurane inhalation to monitor body temperature. The macaques used in this study were free from B virus, hepatitis E virus, *Mycobacterium tuberculosis*, *Shigella* spp., *Salmonella* spp., and *Entamoeba histolytica*.

Vaccines (1 mg/dose) were inoculated subcutaneously into macaques using syringes twice with a two-week interval between injections under ketamine/xylazine anesthesia. Saline instead of the vaccine was inoculated into macaques as unvaccinated controls. The macaques were challenged with Narita1 (2×10^5^ TCID_50_/1 ml) into nasal cavities (0.5 ml for each nostril) with pipettes 7 weeks after the second vaccinations under ketamine/xylazine anesthesia. Experiments using Narita1 were performed in the biosafety level 3 facility of the Research Center for Animal Life Science, Shiga University of Medical Science.

Under ketamine/xylazine anesthesia, 2 cotton sticks (TE8201, Eiken Chemical, Ltd., Tokyo, Japan) were used to collect fluid samples in nasal cavities and tracheas, and the sticks were subsequently immersed in 1 ml of PBS containing 0.1% bovine serum albumin (BSA) and antibiotics.

### Viruses and Vaccines

We used influenza virus A/swine/Hokkaido/2/1981 (H1N1) (National Center for Biotechnology Information (NCBI) taxonomy database ID: 387253) as a vaccine strain [48].

The Hokkaido2 virus was propagated in allantoic cavities of 10-day-old embryonated hen’s eggs at 35°C for 48 h. To prepare an inactivated whole particle vaccine, the allantoic fluid infected with Hokkaido2 was concentrated and purified by high-speed centrifugation (112,500 g for 90 min) through a 10–50% sucrose density gradient and then treated in 0.1% formalin at 4°C for one week [9]. For preparation of a split vaccine, ether was mixed for 30 min with a suspension of the viral particles purified and inactivated by the same method as that for preparation of the whole particle vaccine. Thereafter, ether was evaporated with bubbing at room temperature without removal of hydrophilic solution. The amount of the whole particle vaccine and that of the split vaccine were indicated as the amount of entire proteins including HA and other viral proteins. With an HA test, we confirmed that the two types of vaccine included the same amounts of HA.

Pandemic influenza virus A/Narita/1/2009 pdm (NCBI taxonomy ID: 645520) was used as a challenge virus (kindly provided by Dr. Takato Odagiri, National Institue of Infectious Disease (NIID), Japan) [22]. In a preliminary study, we confirmed that Narita1 inoculated into nostrils, oral cavities and tracheas caused pneumonia in cynomolgus macaques as severe as that observed in macaques inoculated with A/California/04/2009 (H1N1) (NCBI taxonomy ID: 641501) [2]. Narita1 was propagated in embryonated eggs twice at NIID and once in Madin-Darby canine kidney (MDCK) cells (the American Type Culture Collection, Manassas, VA) at the Shiga University of Medical Science [49]. The amino acid sequence identities between Hokkaido 2 and Narita1 were 89% in HA (GI: 216409430 vs. GI: 237659680) and 83% in NA (GI: 216409434 vs. GI: 23761745).

In order to assess virus replication, serial dilutions of swab samples were inoculated onto confluent MDCK cells as described previously [9]. Cytopathic effects were examined under a microscope 72 h later.

### Detection of Antibody Specific for Virus Antigen with ELISA

The antibody titers of serum and swab samples against Hokkaido2 antigens were determined using ELISA [50]. Ninety-six-well plates were coated with 50 µl of purified Hokkaido2 (20 µg/ml) disrupted with 0.05 M Tris-HCl (pH 7.8) containing 0.5% Triton X-100 and 0.6 M KCl. Serially diluted samples were incubated overnight in the coated plates. After washing five times, horseradish peroxidase (HRP) -conjugated anti-monkey IgG antibodies (MP Biomedicals, Inc./Cappel, Aurora, OH) (1∶2000×50 µl), anti-monkey IgA antibodies (Nordic Immunological Laboratories, Tilburg, The Netherlands) (1∶4000×50 µl) or IgM (Rockland Inc., Gilbertsville. PA) (1∶5000×50 µl) were added and incubated for 1 h at room temperature. For detection of IgG1, incubation with anti-human IgG1 (clone: HP6069, Calbiochem) (1∶2000×50 µl) was followed by incubation with HRP-conjugated anti-mouse Ig (Bio-Rad Laboratories, Inc.) (1∶2000×50 µl). HRP activity was assessed using 3, 3′, 5, 5′-tetramethyl benzidine substrate (100 µl). The reaction was stopped by the addition of 1 M hydrogen chloride (100 µl). Optical density was measured at 450 nm.

### Cytokine Assay

In the experiment for which results are shown in Fig. 3, lymphocytes were purified from peripheral blood of the macaques using a density gradient (Wako Pure Chemical Industries Ltd., Osaka, Japan). After washing, CD8^+^ cells were isolated using CD8 microbeads for non-human primates and magnetic cell sorting (MACS, Miltenyi Biotec GmbH, Bergisch Gladbach, Germany), followed by separation of CD4^+^ cells using CD4 microbeads. The cells remaining after removal of CD4^+^ and CD8^+^ cells were used as antigen-presenting cells (APC) after irradiation at 30 Gy. CD4^+^ or CD8^+^ T cells (1×10^5^ cells/well) and APC (0.5×10^5^ cells/well) were cultured with the inactivated whole particle antigens or the ether split vaccine antigen in the presence of anti-CD28 (clone: CD28.2) and CD49d (clone: 9F10) antibodies (0.5 µg/ml, eBioscience Inc., San Diego, CA) in 96-well U-bottom plates for 48 h and supernatants were collected [10]. The concentrations of IFN-γ and IL-2 in the supernatants were measured using the Milliplex MAP non-human primate cytokine panel and Luminex200 (Millipore Corp., Billerica, MA).

In the experiment for which results are shown in Fig. 7C, spleen cells obtained at autopsy on day 14 after the challenge infection were used after homogenization. After washing, CD8^+^ cells were isolated using CD8 microbeads as described above. CD8^+^ T cells (1×10^5^ cells/well) and APC (0.5×10^5^ cells/well) were cultured with various peptides (10 µM) in the presence of anti-CD28 and CD49d antibodies (0.5 µg/ml) in 96-well U-bottom plates for 48 h and supernatants were collected. The concentrations of IFN-γ in the supernatants were measured by ELISA using purified anti-human IFN-γ (clone: MD-1, eBioscience) and biotinylated anti-human IFN-γ (clone: 4S.B3, BioLegend, Inc., San Diego, CA) followed by HRP-labeled streptavidin (BD Biosciences). HRP activity was assessed using 3, 3′, 5, 5′-tetramethyl benzidine substrate as described above.

In the experiment for which results are shown in Fig. 9, unfractionated cells from cervical lymph nodes obtained at autopsy on day 14 after the challenge infection were incubated with NP262-270 peptide (10 µM, Hokkaido System Sciences, Sapporo, Japan) in the presence of anti-CD28, anti-CD49d (0.5 µg/ml) and human IL-2 (10 ng/ml) for 5 d. The cultured lymph node cells were stimulated with phorbol 12-myristate 13-acetate (PMA) (0.1 µg/ml) and ionomycin (1 µg/ml) for 5 h. Monensin (2 µM) was added for the last 4 h. Thereafter, cells were washed with PBS containing EDTA (0.5 µM). Surface CD8 was stained with fluorescein isothiocyanate (FITC)-conjugated specific antibodies (clone: RPA-T8, eBioscience). Intracellular IFN-γ was stained with phycoerythrin (PE)-conjugated antibody (clone: 4S.B3, BD Biosciences) after fixation with 4% paraformaldehyde and permeabilization with 0.1% saponin.

### Virus Neutralization Assay

Serum samples were pretreated with a receptor-destroying enzyme (RDEII, Denka Seiken, Tokyo, Japan) at 37°C overnight and then inactivated at 56°C for 1 h. The diluted samples were mixed with 50 TCID_50_ of the viruses for 1 h. Then the mixture was added onto an MDCK monolayer. After 1-h incubation, the cells were cultured in MEM containing 0.1% BSA and 5 µg/ml trypsin. After incubation at 35°C for 3 days, the number of wells with cytopathic effects was counted in quadruplicate culture. Neutralization titers were expressed as the dilution in which cytopathic effects were observed in 50% of the wells.

### Typing of Mafa-A1 and Transfection of the Mafa-A1*052∶02 Gene

Blood was collected from cynomolgus macaques and immediately mixed with Trizol (Invitrogen Corporation, Carlsbad, CA). After extracting total RNA, cDNA was synthesized using oligo dT primer and ReverTra Ace (Toyobo Co. Ltd., Osaka, Japan). To amplify Mafa-A genes, PCR was performed using a set of primers (each 0.5 µM), sense 5′-GATGGCTATCATGGCGCC-3′ and anti-sense 5′-TCTTCATGCCTTCTCTTTGTGACT-3′, and KOD FX polymerase (0.4 units, Toyobo) [51]. The cycling parameters were as follows: an initial denaturation at 98°C for 1 min followed by 30 cycles of 98°C for 10 s and 68°C for 1 min. The amplified DNA fragments were cloned into pGEM-T Easy vector (Promega, Madison, WI). Thereafter, nucleotide sequences were determined using an ABI3130 genetic analyzer (Applied Biosystems, Foster City, CA) in accordance with the protocol of the Big Dye terminator method.

The Mafa-A1*052∶02 (GenBank AM943361, nomenclature based on the WHO Nomenclature Committee for Factors of the HLA System) gene was inserted as an EcoRI DNA fragment into the EcoRI site of a pTA2 vector (Toyobo) and then cloned into EcoRI sites of a pcDNA3.1^+^ vector (Invitrogen). The Mafa-A1*052∶02 gene cloned in pcDNA3.1 was transfected into RMA-S-expressing human *β2-microglobulin* gene (kindly provided by Dr. Masanori Matsui) using Nucleofector Solution T and a Nucleofector I device (Lonza Group Ltd., Basel, Switzerland) in accordance with the manufacturer’s instructions. Cells that stably expressed the introduced genes were selected with G418 (0.5 mg/ml) and hygromycin B (0.35 mg/ml).

### Peptide Binding Assay

Overlapped peptides based on the amino acid sequence of Narita1 nucleoprotein (GenBank GQ169303, protein ID ACR20063.1) were synthesized by the Sigma Pepscreen system (Sigma-Aldrich Co., St. Louis, MI). Each peptide shared 5 amino acids with consecutive peptides. RMA-S-expressing human *β2-microglobulin* and Mafa-A1*052∶02 genes were cultured overnight at 27°C [52], [53]. Then the cells were washed twice with PBS. RMA-S (5×10^5^ cells/tube) was incubated with each peptide for 30 min at 27°C and then for 4 h at 37°C. Surface Mafa-A1*052∶02 molecules were detected by biotinylated W6/32 and PE-conjugated streptavidin (eBioscience). Dead cells were excluded by using propidium iodide. Expression of Mafa-A1*052∶02 on the cell surface was analyzed using a flow cytometer.

### Establishment of 721.221 Transfected with Mafa-A1*052∶02

pcDNA 3.1^+^ vector containing Mafa-A1*052∶02 gene was introduced into an MHC class I-deficient human B cell line, 721.221 (kindly provided by Dr. Masanori Matsui), by electroporation [54]. Two µg of pcDNA 3.1^+^- Mafa-A1*052∶02 was added to 721.221 (2×10^6^ cells) in 100 µl of Nucleofector™ Solution T and electroporated using program A-24 on a Nucleofector I device. G418-resistant cells were isolated.

### Collection of Peptides from Mafa-A1*052∶02 and LC-MS/MS Analysis

Mafa-A1*052∶02-associated peptides were purified using a modification of a previously described protocol [21]. Briefly, one hundred sixty 75-cm^2^ flasks (BD Biosciences) of 721.221 transfectants expressing Mafa-A1*052∶02 were collected and washed twice with PBS. The cells were resuspended in ice-cold homogenization buffer (250 mM sucrose, 20 mM HEPES-NaOH pH 7.5). The cells were homogenized using a tight-fitting glass dounce homogenizer. The homogenate was spun at 1,000 g for 7 min at 4°C and the supernatant was retained. The pellet was further washed twice with homogenization buffer and the supernatants were added to the first harvest. After centrifugation at 12,000 g for 30 min at 4°C, the pellet was resuspended in lysis buffer (150 mM NaCl, 20 mM Tris-HCl pH 8.0, 1% (v/v) CHAPS solution containing protease inhibitor Cocktail (Roche Ltd.)) for 1 h on ice. After centrifugation at 30,000 g for 1 h at 4°C, the supernatant was immunoaffinity purified with 1.7 mg of purified antibody W6/32 coupled with 1 ml of CNBr-activated Sepharose 4B (GE Healthcare UK Ltd., Buckinghamshire, UK) following the manufacturer’s protocol. Bound peptides were eluted from Mafa-A1*052∶02 with 0.2 M acetic acid (pH 2.7) and were then filtered through a Centricon 10 kDa YM-10 membrane (Millipore Corp.) at 3,500 g for 5 h at 4°C. The filtered sample was concentrated by a vacuum centrifuge, Savant SpeedVac SC-100A (Thermo Fisher Scientific Inc., Waltham, MA), and resuspended in a solution consisting of 2% acetonitrile, 0.1% trifluoroacetic acid and 98% water. The sample was injected for LC-MS/MS analysis.

A liquid chromatography system, Paradigm MG4 (AMR Inc., Tokyo, Japan), was coupled online to a linear ion trap mass spectrometer (LTQ, Thermo Electron Corp., Waltham, MA) equipped with an electrospray interface operated in positive ion mode. All MS/MS spectra were identified using SEQUEST (v.28 (revision 12), Thermo Electron Corp.).
